# Association between Gait Variability and Gait-Ability Decline in Elderly Women with Subthreshold Insomnia Stage

**DOI:** 10.3390/ijerph17145181

**Published:** 2020-07-17

**Authors:** Taesang Lee, Myeounggon Lee, Changhong Youm, Byungjoo Noh, Hwayoung Park

**Affiliations:** 1 Major in Dance Division of Creative Performing Arts, Silla University, Sasang-gu, Busan 46958, Korea; its73@silla.ac.kr; 2 Biomechanics Laboratory, College of Health Sciences, Dong-A University, Saha-gu, Busan 49315, Korea; freestyle710@naver.com (M.L.); app00113@dau.ac.kr (H.P.); 3 Department of Healthcare and Science, College of Health Sciences, Dong-A University, Saha-gu, Busan 49315, Korea; bnoh@dau.ac.kr

**Keywords:** sleep quality, menopause, dynamic stability, inertial measurement units (IMU), walking speed, gait variability

## Abstract

This study investigates the gait characteristics of elderly women, aged more than 65 years, with subthreshold insomnia stage at various walking speeds. A total of 392 participants (insomnia: 202 and controls: 190) wearing shoe-type inertial measurement units completed walking tests on a treadmill for a duration of 1 min at slower, preferred, and faster speeds. The insomnia group indicated lower pace parameters (range of Cohen’s *d*: 0.283–0.499) and the single support phase (Cohen’s *d*: 0.237), greater gait variability (range of Cohen’s *d*: 0.217–0.506), and bilateral coordination (range of Cohen’s *d*: 0.254–0.319), compared with their age-matched controls; the coefficient of variance (CV) of the stance phase at the faster speed condition was a crucial variable for distinguishing between insomnia and control groups. In addition, the insomnia group demonstrated insufficient gait adaptation at the slower and preferred speeds, as indicated by the CVs of the stride length, stride time, and step time. In particular, participants with worsened insomnia symptoms or sleep problems showed that these worse gait patterns may increase the potential risk of falling in elderly women. Thus, elderly women with subthreshold insomnia stage need to improve their sleep quality to enhance their physical functions.

## 1. Introduction

Gait, which is a task involving forward movement and maintenance of stability during walking, is one of the fundamental motor functions in humans [1]. Gait analysis has been performed to evaluate various health-related factors such as physical functions, health status, and quality of life, as well as disease progression [2,3]. In addition, it has been used to investigate the effect of gait disturbances on healthy individuals as well as pathological patients [3]. For instance, a slower walking speed indicates the decline of gait stability in older adults [3,4,5,6]. In addition, gait variability (GV) [7,8] and the parameters of bilateral coordination, such as gait asymmetry (GA) and phase coordinate index (PCI) [9,10], are used to evaluate dynamic stability during gait tasks. Many studies have reported that slower walking speed and greater GV may indicate the decline of gait ability [3,4,5,6,7,10], which may increase the risk of falling in elderly adults.

Insomnia is defined as a sleep dissatisfaction, either qualitatively or quantitatively [11,12]. It is the most common sleep disorder, occurring in 6–18% of the general population [13]. Insomnia is common in elderly adults [7] in particular, with an overall prevalence of 30–48% [14,15]. Insomnia symptoms, such as difficulty in initiating sleep, difficulty in maintaining sleep due to frequent awakening, or problems returning to sleep after awakening, and early-morning awakening with an inability to return to sleep [11], result in lesser sleep time and poorer sleep quality [12]. These insomnia symptoms may also be associated with mental disorders such as depression, anxiety [11,12], and cognitive function [11,16].

The previously reported significant factors related to insomnia include gender, age, socioeconomic status, and psychiatric comorbidities [15]. The number of menopausal elderly women suffering from sleep disturbances or insomnia is higher, compared to the number of elderly men of the same age, due to estrogen hormone deficiency [12]. Physical activity (PA) can reduce insomnia symptoms and enhance sleep quality [17,18]; however, menopausal elderly women have muscle weakness, which may lead to reduced PA [19,20]. In addition, previous studies reported insomnia symptoms and sleep disturbances are associated with the falling risk in elderly adults [21,22]. Thus, elderly women with insomnia may impose a potential societal cost [12,23], which may decrease the quality of life.

Insomnia may also affect the gait stability in older adults, because it may indicate a poor sleep quality [11]; Agmon et al. [24] reported that poor sleep quality in older adults decreased the walking speed and increased GV values during dual tasks, indicating a potential risk of falling in elderly adults. However, they suggested further studies to elucidate the relationship between sleep quality and gait characteristics. Participants who faced the subthreshold insomnia stage are not clinical insomnia patients, but they may suffer from poor sleep quality and sleep problems. Thus, the evaluation of gait ability with respect to subthreshold insomnia should be utilized as a reference to mitigate falling risks and implement intervention programs to enhance gait ability in elderly women.

Moreover, most previous studies used averaged data through repeated trials to collect multiple steps (not continuous steps); however, with this method, it may be difficult to replicate the natural walking patterns of individuals [25]. Recently, a significant decline in gait ability has been reported, based on the quantitative speed value range (e.g., ±20% of the individual’s preferred walking speed); this speed value range has been validated in healthy young adults [26], elderly adults [27], and Parkinson’s patients [28]. Thus, gait stability at slower, faster speeds, and self-preferred speeds may be beneficial to understanding the gait characteristics in elderly women with subthreshold insomnia.

In view of the above, this study investigated gait characteristics in elderly women aged over 65 years in the subthreshold insomnia stage under various conditions, such as slower, preferred, and faster speeds. We hypothesize that our insomnia group, which exhibited a subthreshold insomnia stage, would exhibit reduced gait stability, indicated by a higher GV and bilateral coordination at slower or faster speed conditions. In addition, we hypothesize that this group would exhibit an insufficient gait adaptation ability with an increase in walking speed.

## 2. Materials and Methods

### 2.1. Study Population

**Figure 1:**
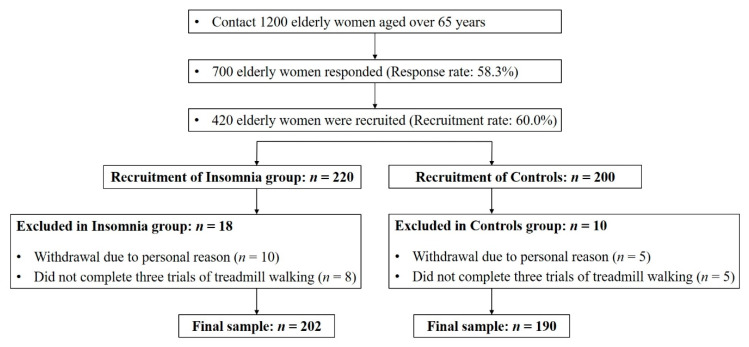
Recruitment process flowchart.

Participants were recruited as part of a community-wide survey in Busan metropolitan city from February to December 2019. We contacted 1200 elderly women over 65 years living in the community, and 700 people responded (response rate: 58.3%). Eventually, we recruited 420 participants (insomnia group: 220, controls: 200) for this study (recruitment rate: 60.0%). Twenty-eight participants were excluded from the study, because they either could not complete the three treadmill test trials (*n* = 13) successfully or withdraw for the personal reasons in the test day (*n* = 15). In total, 392 participants (insomnia: 202, controls: 190) successfully performed the three treadmill trial walking tests (Figure 1 and Table 1). None of the participants had a history of musculoskeletal or neurological problems that affect gait, and all could walk without support. All the participants read and signed an informed consent form approved by the Institutional Review Board of Dong-A University (IRB number: 2-1040709-AB-N-01-201901-HR-011-02). Moreover, this study was carried out in accordance with the Declaration of Helsinki and the ethical guidelines for human subjects of the Institutional Review Board of Dong-A University.

The severity of insomnia was evaluated using an insomnia severity index (ISI) questionnaire, which had seven questions assessing the severity of sleep onset, sleep maintenance difficulties, and satisfaction with current sleep [29]. The ISI can be used as a screening measure in clinical practice, which allows for the assessment of the change following treatment, and is also useful for epidemiological research [29,30]. Each answer was scored on the Likert scale, and included none (score: 0), mild (score: 1), moderate (score: 2), severe (score: 3), and very severe (score: 4). The seven answers were added to obtain the total score, which was used to determine the severity of insomnia: (1) 0–7: no sustained insomnia; (2) 8–14: subthreshold insomnia; (3) 15–21: clinical insomnia at moderate severity; and (4) 22–28: severe clinical insomnia [29,31]. Participants in the subthreshold insomnia stage were classified as the insomnia group (ISI total score: below 14) in this study.

### 2.2. Instrumentation

Shoe-type inertial measurement unit (IMU) system (DynaStab^TM^, JEIOS, Busan, Korea) is a gait analysis system comprising shoe-type data loggers (Smart Balance SB-1^®^, JEIOS, Busan, Korea), including an IMU sensor (IMU-3000^TM^, InvenSense, San Jose, CA, USA) installed on the outsoles of both shoes and data acquisition and analysis system (Smart Balance 1.5 version, JEIOS, Busan, Korea), were utilized in this study [32,33]. The accelerations and angular velocities data were transmitted wirelessly to a data acquisition system through Bluetooth^®^ [32,33]. The shoe-type IMU system indicated an excellent agreement between the recorded data from the healthy adults [32] and Parkinson’s patients [33] (intra-class coefficient value: 0.97–0.99). The shoe sizes were adapted to fit the participants, and a range of shoe sizes was available (225–280 mm). The speed of the belt on the treadmill (HK-365, Healthkeeper, Seoul, Korea) can be controlled in the range of 0.5–16 km/h at increments of 0.1 km/h.

### 2.3. Test Procedure

All the test procedures, such as the measurement of the demographic characteristics, questionnaires, and gait tasks, were completed in a single day. The biometric data of all the participants, such as body height, weight, and body fat percentage, were recorded before the treadmill walking test. All the participants completed questionnaires to assess their ISI response, global cognitive function, PA, and stress response. The mini-mental state examination (MMSE) questionnaire was used to assess global cognitive function [34], and PA was evaluated using the international PA questionnaire–short form (IPAQ-SF). The IPAQ-SF was composed of seven items on the self-reported PAs of the participants, which evaluate the level of PA by vigorous, moderate, lower, and walking PAs. We calculated the metabolic equivalents (METs)/week for vigorous, moderate, walking, and total PAs based on the IPAQ-SF [35]. The stress response was assessed using the modified stress response inventory (SRI-MF) comprising 22 questions, each of which was scored on the Likert scale and included “not at all”, “somewhat”, “moderately”, “very much”, and “absolutely” [36]. These 22 questions were categorized into three simplified stress factors, namely somatization, depression, and anger; Cronbach’s alphas for the SRI [37] were indicated by somatization (0.89), depression (0.88), and anger (0.87), respectively [36]. A higher SRI-MF total score indicated a severe stress level.

**Figure 2:**
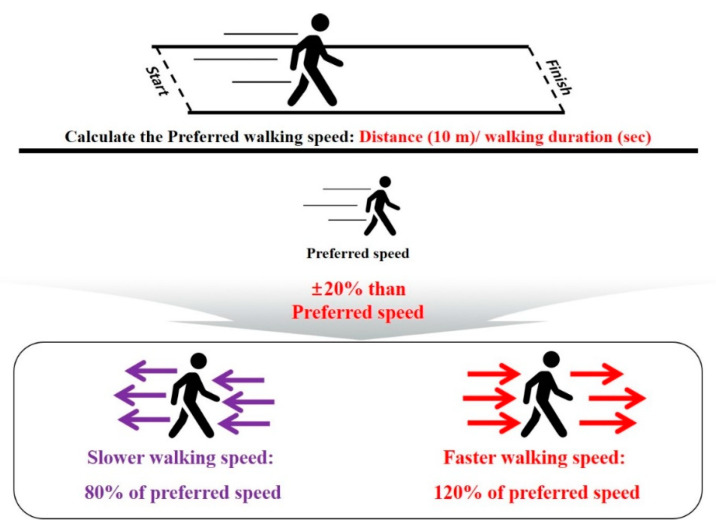
Walking speed definitions: slower (20% less than the preferred) and faster (20% more than the preferred) speeds, calculated based on the participant’s preferred speed.

Before the treadmill test, all the participants completed overground walking tests in a straight 10-m walkway, to calculate their self-preferred walking speed (distance/walking duration) (Figure 2). Slower (80% of preferred speed) and faster (120% of preferred speed) speeds were calculated relative to the preferred speed [27,28]. For instance, if the measured preferred speed was 1.0 m/s, then slower and faster speeds are 0.8 m/s and 1.2 m/s, respectively. Subsequently, the participants practiced the treadmill walking exercises until they were comfortable with their self-preferred speed, as well as the slower and faster speed conditions. After practicing for speed adaptation, the participants took a rest of about 5 min. During the treadmill walking test, the participants were asked to walk to maintain a stable walking pattern for each speed condition on the treadmill for about 30–60 s at the onset of the treadmill walking. An operator collected the treadmill walking data at 100 Hz (1-min periods) when a participant attained a stable walking pattern [38].

### 2.4. Data Analyses

The treadmill walking data were filtered using a second-order Butterworth low pass filter, and a cutoff frequency was set at 10 Hz [32,33]. The gait events were defined as follows: (a) heel strike when the linear acceleration on the anteroposterior axis reached its maximum value, and (b) toe-off at instances in which the linear acceleration on the vertical axis reached its maximum value during the gait cycle [32,39].

The spatiotemporal parameters were calculated as follows: (1) pace: walking speed, stride length, and step length; (2) rhythm: cadence, stride time, and step time; (3) phases: single support, double support, and stance. As the GV case, the percentage coefficient of variance (CV) values were calculated for all the spatiotemporal parameters ((standard deviation/mean) × 100) [40,41].

To evaluate bilateral coordination, we calculated GA and PCI based on a previous study [9]. GA is an index for the temporal symmetry between the left and right foot during walking. The PCI value was calculated based on a combination of the percentage_ABS_φ and CV of φ [9]. The increased GA and PCI values indicate reduced bilateral coordination, which may suggest a worsened gait stability [9,42]. Essential definitions of the gait-related variables are shown in Table 2. The analyzed data are presented in Table S1.

### 2.5. Statistical Analyses

The Shapiro–Wilk test was used to determine whether the data were normally distributed. An independent sample *t*-test was used to compare the differences between the insomnia group and the controls (*p* < 0.05). A one-way repeated measures analysis of variance with Bonferroni correction (*p*-value: 0.05/3, 0.0167) was used to compare the differences between the slower, preferred, and faster speed conditions.

In addition, a binary logistic regression analysis using the forward and backward stepwise method was performed to determine the classifiers between insomnia and control groups based, on the gait-related variables. Prior to the logistic regression analysis, we conducted a univariable logistic analysis, to minimize the multicollinearity problems. Then, a multivariable stepwise binary logistic regression analysis was performed for each speed condition. The model was adjusted for age, BMI, and body fat percentage. Furthermore, after determining the relationships between insomnia and functional and gait characteristics, Pearson’s product-moment correlation analysis was used, to compare between the total ISI score and functional characteristics, such as the global cognitive function (MMSE), PA (IPAQ-SF), and stress response (SRI-MF), as well as the gait-related variables for all the participants. All statistical analyses were performed using a statistics software program for Windows (version 21.0, SPSS Inc., Chicago, IL, USA). The statistical significance was set to 0.05.

## 3. Results

### 3.1. Group Differences: Insomnia vs. Control Group

The insomnia group exhibited significantly lower pace parameters for walking speed (slower speed, Cohen’s *d* = 0.499; preferred speed, Cohen’s *d* = 0.455; faster speed, Cohen’s *d* = 0.427), stride length (slower speed, Cohen’s *d* = 0.283; preferred speed, Cohen’s *d* = 0.351; faster speed, Cohen’s *d* = 0.351), and step length (slower speed, Cohen’s *d* = 0.283; preferred speed, Cohen’s *d* = 0.345; faster speed, Cohen’s *d* = 0.358), and higher GV parameters for phases of single support (slower speed, Cohen’s *d* = 0.229; preferred speed, Cohen’s *d* = 0.371; faster speed, Cohen’s *d* = 0.395), double support (slower speed, Cohen’s *d* = 0.247; preferred speed, Cohen’s d = 0.459; faster speed, Cohen’s *d* = 0.411), and stance (slower speed, Cohen’s *d* = 0.217; preferred speed, Cohen’s *d* = 0.427; faster speed, Cohen’s *d* = 0.476), and bilateral coordination for GA (slower speed, Cohen’s *d* = 0.259; preferred speed, Cohen’s *d* = 0.310; faster speed, Cohen’s *d* = 0.304) and PCI (slower speed, Cohen’s *d* = 0.254; preferred speed, Cohen’s *d* = 0.319; faster speed, Cohen’s *d* = 0.304), compared with the controls at all the speed conditions. In addition, the insomnia group exhibited significantly lower phase parameters, including the double support phase (preferred speed, Cohen’s *d =* 0.239, *p* < 0.001; faster speed, Cohen’s *d* = 0.260, *p* = 0.009) and stance phase (preferred speed, Cohen’s *d* = 0.235, *p* < 0.001; faster speed, Cohen’s *d* = 0.241, *p* = 0.017), compared with the controls. However, they exhibited higher GV for the stride length (preferred speed, Cohen’s *d* = 0.376, *p* < 0.001; faster speed, Cohen’s *d* = 0.410, *p* < 0.001), step length (preferred speed, Cohen’s *d =* 0.506, *p* < 0.001; faster speed, Cohen’s *d =* 0.441, *p* < 0.001), stride time (preferred speed, Cohen’s *d* = 0.376, *p* < 0.001; faster speed, Cohen’s *d* = 0.410, *p* < 0.001), and step time (preferred speed, Cohen’s *d* = 0.447, *p* < 0.001; faster speed, Cohen’s *d* = 0.416, *p* < 0.001). The single support phase for the insomnia group was significantly different compared with that of the control group, only at the faster speed condition (Cohen’s *d* = 0.237, *p* = 0.019) (Table 3).

### 3.2. Speed Differences: Slower, Preferred, and Faster Speed Condition Gait-Related Variables for All the Participants

In the insomnia group, the pace, rhythm, phase GV (step length, phases for the single support, double support, and stance), and bilateral coordination parameters were significantly different at the slower, preferred, and faster speed conditions. In addition, the GVs for the stride length, stride time, and step time were significantly different between the slower and faster, and the preferred and faster speed conditions. In the control group, all the gait-related variables were significantly different at the slower, preferred, and faster speed conditions (Table 3).

### 3.3. Classifier Variables for the Insomnia and Control Groups

Stepwise binary logistic regression analysis for the insomnia and control groups revealed that the walking speed was significantly different at the slower speed condition (odds ratio [OR]: 0.024, 95% confidence interval [CI]: 0.005–0.119, *p* < 0.001). At the preferred speed condition, the walking speed (odds ratio [OR]: 0.124, 95% confidence interval [CI]: 0.030–0.505, *p* = 0.004) and CV of the double support phase (odds ratio [OR]: 1.070, 95% confidence interval [CI]: 1.019–1.123, *p* = 0.006) were significantly different between the two groups. At the faster speed condition, the walking speed (odds ratio [OR]: 0.209, 95% confidence interval [CI]: 0.060–0.729, *p* = 0.014) and CV of the stance phase (odds ratio [OR]: 1.229, 95% confidence interval [CI]: 1.042–1.451, *p* = 0.014) were significantly different between the two groups (Table 4).

### 3.4. Relationship between the Total ISI Score and Functional Characteristics for the Global Cognitive Function, PA, and Stress Responses of All the Participants

The total ISI score was negatively correlated with the MMSE score (r = −0.196, *p* < 0.05) and the total PA (r = −0.108, *p* < 0.05), whereas it was positively correlated with the total SRI-MF score (r = 0.224, *p* < 0.05).

### 3.5. Relationship Between the Total ISI Score and Gait-Related Variables of all the Participants

The total ISI score was negatively correlated with pace parameters, such as the walking speed, stride length, and step length, whereas it was positively correlated with GA at all the speed conditions. The phase parameters, including the double support phase and stance phase, were significantly correlated with the total ISI score at a faster speed. The GV of the pace, phase, and rhythm, bilateral coordination of PCI, and CVs of the stride length, step length, single support phase, double support phase, stance phase, stride time, and step time were positively correlated with the total ISI score at the preferred and faster speed conditions (Table 5).

## 4. Discussion

The main findings of this study are as follows: (1) the insomnia group exhibited lower pace parameters and the single support phase, and greater GV and bilateral coordination than the age-matched controls; (2) the CV of the stance phase at the faster speed condition was a crucial variable for distinguishing the insomnia group from the controls; (3) the insomnia group demonstrated insufficient gait adaptations within slower and preferred speeds, as indicated by the CVs of the stride length, stride time, and step time; (4) a higher total ISI score was associated with declined gait ability, as indicated by the increased GV parameters and bilateral coordination. These findings are discussed in detail below.

Elderly menopausal women are deficient in sex hormones, such as estrogen, which may cause muscle weakness [19,20]. This factor may contribute to a decline in their physical performances, walking speed, and PA [23]. Moreover, a lower PA level may affect the gait ability of the insomnia group, because PA can improve gait stability [43,44,45]. Our insomnia group indicated relatively worse PA and stress levels compared to their age-matched controls, and the total ISI score was negatively correlated with the total PA (*r* = −0.103), whereas it was positively correlated with the SRI-MF (*r* = 0.224). These results may indicate that elderly women with a subthreshold insomnia stage may suffer from higher stress levels and gait ability deterioration compared to their age-matched controls. Furthermore, our insomnia group demonstrated reduced gait stability, indicated by the decrease in the pace domain and single support phase (phase domain); the GV parameters and bilateral coordination were more than those of the controls at most speed conditions. Slower walking speed can contribute to longer double support and stance phases, and a shorter single support phase [1]. Similar gait patterns are commonly observed in elderly adults. Previous studies reported increased step widths and double support phases, to enhance the dynamic stability of elderly adults during walking. However, these changes evoked longer stance phases in response to the reduced lower-limb strength [4,5,6,27]. These gait patterns also contributed to an increase in the GV parameters and coordination of the left-right leg movement during walking [9,10,42], including GA and PCI values, indicating a decline in gait stability [7]. Consequently, our insomnia group exhibited reduced gait ability, compared with the controls at all the speed conditions.

Interestingly, the result of a logistic analysis indicated that the CV of the stance phase at a faster speed was approximately 22.9% greater in the insomnia group, compared with the controls, which is possible because the faster walking speed condition was a more challenging task for participants with subthreshold insomnia stage. Slower or faster speed conditions when an individual performs gait tasks may reduce gait automaticity with higher attention-demanding motor cortex controls than the preferred speed condition [7]. Reduced-automaticity gait patterns rely on executive functions requiring more cognitive load, leading to slower processing and alteration in the gait pattern [24,46,47,48]. Although healthy elderly participants performed the gait tasks successfully, they indicated greater GV than young adults, owing to physiological changes caused by increased neuromotor noise [5]. A similar study reported that Parkinson’s patients exhibited 2.1 times (110%) greater CV for the double support phase, compared with their age-matched elderly controls at a faster speed; it was suggested that this result might be related to the control of automatized behavior [28].

In addition, the symptoms of insomnia may deteriorate cognition impairments [11,12,16,49], because poor sleep quality may be related to cortical atrophy in elderly adults [11,50]. Our insomnia group indicated relatively greater ISI and lower MMSE scores (significant but small difference) compared to their age-matched controls, and the results of the correlation analysis indicated that the total ISI score was negatively correlated with the MMSE score in this study. These results may indicate that the elderly adults with subthreshold insomnia stage may be exposed to the possibility of a deteriorated cognitive function. In addition, Agmon et al. [24] reported that lower sleep quality was associated with increased GV in elderly adults during dual tasks, owing to neuro-connections between the sleep-regulation nuclei and the regions controlling gait, including the pontine tegmentum, pedunculopontine nucleus, and medial medulla [24,51]. Hence, gait tasks at the faster speed condition may require more cognitive load, as well as control of motor function in the insomnia group. Therefore, although participants in our insomnia group were not clinical patients, but only had insomnia symptoms in the subthreshold stage, they exhibited reduced gait stability at the faster speed condition, and the GV for the stance phase at the faster speed condition may be a classifier between the insomnia group and controls.

Our insomnia group exhibited a decreasing trend at the slower and preferred speed conditions; however, there were no significant differences in the CVs of the stride length, stride time, and step time, whereas the controls exhibited significant differences at these two-speed conditions. These patterns may be one of the symptoms of declining gait stability in the insomnia group; walking with a self-preferred speed minimizes individual energy cost, which may contribute to maintaining the minimum GV during gait [7,52]. It has been previously reported that control of the walking-related rhythmic stepping mechanism is reflected by the stride time and stride length variability [53], and is mainly dependent on the basal ganglia and spinal central pattern generator [54]. Thus, low stride-to-stride variability reflects automatic processes that require minimal attention, and is associated with efficient gait control and gait safety [55,56,57]. However, poor sleep quality led to an increase in GV values [24], and insomnia and sleep problems are attributed to the potential falling risks in elderly adults [21,22]. Thus, our insomnia group may exhibit reduced gait adaptation ability under the conditions of slower and preferred speeds, which increased the risk of falling.

We observed that the total ISI score was negatively correlated with pace parameters, such as the walking speed, stride length, and step length at all the speed conditions; however, it was positively correlated with the phase parameter for the double support and stance phases at the faster speed condition. In addition, the total ISI score was positively correlated with GV parameters such as the pace (CVs of the stride length and step length), phase (CVs of the single support, double support, and stance phases), and rhythm (CVs of the stride time and step time), as well as bilateral coordination for PCI at the preferred and faster speed conditions. GA was positively correlated with the total ISI score at all the speed conditions. These results indicate that participants with insomnia symptoms may be associated with reduced gait ability because of the increase in GV and bilateral coordination; Agmon et al. [24] also reported that lower sleep quality decreases the motor-cognitive function, increasing GV. In addition, significant trends for GV parameters and bilateral coordination were observed at the preferred (*r*-value range: 0.116–0.206) and faster (*r*-value range: 0.105–0.185) speed conditions, and the MMSE score indicated negative correlation with the total ISI score. It may be possible that the walking task at the preferred and faster speed may be related to the characteristics of gait patterns or relatively difficult tasks owing to reduced gait automaticity [7,24,46,47] in participants with insomnia symptoms. Consequently, these gait patterns may increase the risk of falling in elderly adults [24]. Therefore, we suggest that even the elderly women in the subthreshold insomnia stage may contribute to the decline in their gait ability; in particular, participants with worsened insomnia symptoms or sleep problems showed that these worse gait patterns may increase the potential risk of falling in elderly women.

Our study determined that insomnia symptoms were related to the decline in the gait stability of elderly women, even though they were not clinical patients. However, this study has several limitations. Our study used questionnaires to evaluate the severity of insomnia and PA. However, this method is a subjective measurement method, and may provide inaccurate information, even after valid assessment [13]. The objective measurement techniques using an accelerator can evaluate the PA more accurately, and, using polysomnography (PSG), can provide reliable data, which may be useful in understanding the characteristics of elderly women with insomnia symptoms in detail. In addition, although our study was cross-sectional, long-term studies are needed (e.g., a cohort study) to establish the trend of the relationship between sleep quality and gait ability in elderly women. Furthermore, our study did not consider the effects of the essential characteristics of elderly adults like frailty status, drug dosage and fear of falling using specific questionnaires. Thus, future studies need to consider the relationships between these factors and the gait ability in elderly women with insomnia symptoms. Finally, our walking tasks at the slower, preferred, and faster speed conditions were conducted on a treadmill for a duration of 1 min, which is useful for collecting numerous continuous walking steps [58]. However, treadmill walking tasks may indicate relatively less GV values compared with overground walking, according to the task characteristics, because the walking speed on the treadmill imposes a steady speed condition, which minimizes the variance of the step compared with overground walking [38]. In addition, the treadmill walking speed acts as an external cue for the participants [7]. Nevertheless, all the participants successfully completed the treadmill walking tasks at all the speed conditions (except 13 people), and this may be utilized as a reference in gait training programs.

## 5. Conclusions

This study investigated the gait characteristics at various conditions such as slower, preferred, and faster speeds in elderly women aged more than 65 years with subthreshold insomnia stage. We found that individuals with subthreshold insomnia stage exhibited lower gait ability compared with their age-matched controls, as indicated by the pace, single support phase, GV parameters, and bilateral coordination; the CV of the stance phase at the faster speed condition was a classifier to distinguish between the insomnia and control groups. In addition, the insomnia group demonstrated insufficient gait adaptations at the slower and preferred speeds, compared with the controls, as indicated by the CVs of the stride length, stride time, and step time. Finally, increased insomnia severity was associated with the decline in gait ability, as indicated by the increase in GV parameters and bilateral coordination. Therefore, we suggest that insomnia symptoms may cause a decline in gait ability; in particular, participants with worsened insomnia symptoms or sleep problems may increase the potential risk of falling in elderly women. Thus, elderly women with subthreshold insomnia stage need to improve their sleep quality to enhance their physical functions.

## Figures and Tables

**Table 1:** Demographic characteristics of the study participants.

Variables	Insomnia (*n* = 202) (min to max, 95% CI)	Controls (*n* = 190) (min to max, 95% CI)	*T*-Test *p*-Value	Cohen’s d
Age (years)	71.3 ± 4.6 (70.7 to 72.0)	71.4 ± 4.9 (70.7 to 72.1)	0.876	0.016
Height (cm)	155.5 ± 5.3 (154.7 to 156.2)	154.9 ± 5.9 (154.1 to 155.8)	0.330	0.098
Body weight (kg)	58.8 ± 7.3 (57.8 to 59.8)	59.4 ± 7.9 (58.3 to 60.5)	0.438	0.078
BMI (kg/m^2^)	24.3 ± 2.9 (23.9 to 24.7)	24.8 ± 3.1 (24.3 to 25.2)	0.150	0.147
Body fat percentage (%)	32.9 ± 5.9 (32.1 to 33.7)	33.0 ± 6.6 (32.0 to 33.9)	0.878	0.016
ISI (score)	10.9 ± 2.4 (10.5 to 11.2)	2.0 ± 2.5 (1.6 to 2.3)	<0.001	3.639
MMSE (score)	27.4 ± 2.6 (27.0 to 27.8)	27.9 ± 2.1 (27.6 to 28.2)	0.022	0.233
SRI-MF (score)	5.3 ± 6.3 (4.4 to6.2)	3.1 ± 4.9 (2.4 to 3.8)	<0.001	0.396
Vigorous PAs (Mets/week)	997.8 ± 1385.7 (805.6 to 1190.1)	1118.5 ± 1696.8 (875.7 to 1361.3)	0.440	0.078
Moderate PAs (Mets/week)	618.9 ± 743.1 (515.8 to 722.0)	817.7 ± 951.3 (681.5 to 953.8)	0.021	0.232
Walking PAs (Mets/week)	787.8 ± 927.5 (659.1 to 916.5)	997.7 ± 977.76 (857.8 to 1137.7)	0.030	0.220
Total PAs (Mets/week)	2404.5 ± 2304.1 (2084.8 to 2724.2)	2933.9 ± 2487.9 (2577.9 to 3290.0)	0.029	0.213

Mean ± standard deviation; BMI: body mass index; ISI: insomnia severity index; MMSE: mini-mental state examination; SRI-MF: modified form of stress response inventory; PAs: physical activities; *T*-test: independent *T*-test, *p* < 0.05.

**Table 2:** Definitions of the gait-related variables.

Variables	Definition
Pace parameters	
Walking speed (m/s)	Performed treadmill walking speed
Stride length (m)	Distance between the first contact of one foot to the first contact of the following ipsilateral foot, measured parallel to the direction of progression
Step length (m)	Distance between the successive heel points of opposite feet, measured parallel to the direction of progression
Rhythm parameters	
Cadence (beats/min)	Total number of steps during 1 min
Stride time (s)	Time taken between the first contact of one foot to the first contact of the following ipsilateral foot
Step time (s)	Time taken between the first contact of one foot to the first contact of the following contralateral foot
Phase parameters	
Single support phase (%)	Time when one foot is in contact with the ground during one stride cycle
Double support phase (%)	Time when both feet is in contact with the ground during one stride cycle
Stance phase (%)	Time when foot is in contact with the ground during one stride cycle
Gait variability	
Parameters for pace, rhythm, and phase (%)	Coefficient of variance ((standard deviation/mean) × 100) for stride length, step length, stride time, step time, single support phase, double support phase, and stance phase
Bilateral coordination [9]	
GA (%)	Temporal symmetry between the left and right foot during walking
PCI (%)	Coordination between the left and right foot during walking

GA: gait asymmetry; PCI: phase coordinate index.

**Table 3:** Differences in gait variables in insomnia and control groups at different speeds.

Variables	Slower Speed		Preferred Speed		Faster Speed		Group Significance	Speed Significance	
	Insomnia	Controls	Insomnia	Controls	Insomnia	Controls		Insomnia	Controls
Pace parameters									
Walking speed (m/s)	0.67 ± 0.15	0.74 ± 0.13	0.84 ± 0.19	0.92 ± 0.16	1.01 ± 0.23	1.10 ± 0.19	A, B, C	d, e, f	d, e, f
Stride length (m)	0.90 ± 0.24	0.96 ± 0.18	0.99 ± 0.26	1.07 ± 0.19	1.10 ± 0.26	1.18 ± 0.19	A, B, C	d, e, f	d, e, f
Step length (m)	0.45 ± 0.12	0.48 ± 0.09	0.49 ± 0.13	0.53 ± 0.10	0.55 ± 0.13	0.59 ± 0.09	A, B, C	d, e, f	d, e, f
Rhythm parameters									
Cadence (beats/min)	92.50 ± 17.10	92.78 ± 14.72	103.49 ± 15.41	104.15 ± 14.62	110.66 ± 14.20	111.79 ± 12.97	N/S	d, e, f	d, e, f
Stride time (s)	1.33 ± 0.24	1.32 ± 0.21	1.18 ± 0.18	1.17 ± 0.17	1.10 ± 0.14	1.08 ± 0.13	N/S	d, e, f	d, e, f
Step time (s)	0.67 ± 0.12	0.66 ± 0.10	0.59 ± 0.09	0.58 ± 0.08	0.55 ± 0.07	0.54 ± 0.06	N/S	d, e, f	d, e, f
Phase parameters									
Single support phase (%)	35.17 ± 2.61	35.51 ± 2.12	36.48 ± 2.20	36.85 ± 1.64	37.43 ± 2.03	37.86 ± 1.57	C	d, e, f	d, e, f
Double support phase (%)	29.46 ± 4.79	29.05 ± 4.16	27.09 ± 3.57	26.29 ± 3.10	25.14 ± 3.32	24.32 ± 2.90	B, C	d, e, f	d, e, f
Stance phase (%)	64.63 ± 2.61	64.56 ± 2.37	63.57 ± 1.86	63.14 ± 1.80	62.57 ± 1.65	62.18 ± 1.58	B, C	d, e, f	d, e, f
Gait variability									
CV of stride length (%)	3.45 ± 2.12	3.20 ± 1.74	3.24 ± 2.24	2.53 ± 1.45	2.39 ± 1.79	1.79 ± 1.04	B, C	e, f	d, e, f
CV of step length (%)	2.19 ± 1.54	2.06 ± 1.09	1.94 ± 1.47	1.42 ± 0.76	1.30 ± 1.02	0.95 ± 0.47	B, C	d, e, f	d, e, f
CV of stride time (%)	3.45 ± 2.12	3.20 ± 1.74	3.24 ± 2.24	2.53 ± 1.45	2.39 ± 1.79	1.79 ± 1.04	B, C	e, f	d, e, f
CV of step time (%)	3.31 ± 2.07	3.17 ± 1.72	3.28 ± 2.27	2.45 ± 1.32	2.38 ± 1.90	1.77 ± 0.83	B, C	e, f	d, e, f
CV of single support phase (%)	7.32 ± 3.92	6.55 ± 2.70	5.38 ± 3.22	4.42 ± 1.73	4.06 ± 3.41	3.05 ± 1.19	A, B, C	d, e, f	d, e, f
CV of double support phase (%)	12.35 ± 8.02	10.71 ± 4.85	10.59 ± 7.76	7.80 ± 3.69	8.20 ± 8.06	5.71 ± 2.90	A, B, C	d, e, f	d, e, f
CV of stance phase (%)	5.69 ± 3.67	5.01 ± 2.47	4.63 ± 3.39	3.46 ± 1.87	3.16 ± 2.31	2.28 ± 1.22	A, B, C	d, e, f	d, e, f
Bilateral coordination									
GA (%)	4.57 ± 3.76	3.67 ± 3.15	3.88 ± 3.65	2.87 ± 2.82	3.19 ± 2.92	2.41 ± 2.16	A, B, C	d, e, f	d, e, f
PCI (%)	5.92 ± 4.32	5.05 ± 2.21	5.35 ± 4.75	4.14 ± 2.50	4.72 ± 5.26	3.26 ± 1.85	A, B, C	d, e, f	d, e, f

GA: gait asymmetry; PCI: phase coordinate index. Group differences between insomnia and controls for slower (A), preferred (B), and faster (C) speeds, *p* < 0.05; speed differences within slower vs. preferred (d), slower vs. faster (e), preferred vs. faster (f), *p* < 0.0167 (0.05/3), N/S indicates no significance.

**Table 4:** Binary logistic regression results for insomnia and control groups.

Variables	Estimate	SE	OR	95% CI for the OR	*p*-Value
Slower speed					
Walking speed	−3.740	0.824	0.024	0.005–0.119	<0.001
Preferred speed					
Walking speed	−2.086	0.716	0.124	0.030–0.505	0.004
CV of double support phase	0.068	0.025	1.070	1.019–1.123	0.006
Faster speed					
Walking speed	−1.567	0.638	0.209	0.060–0.729	0.014
CV of stance phase	0.207	0.084	1.229	1.042–1.451	0.014

Model adjusted for age, BMI, and % body fat. CI: confidence interval; OR: odds ratio; SE: standard error.

**Table 5:** Pearson’s product-moment correlation analysis between the total insomnia severity index (ISI) score and gait-related variables of all the participants at slower, preferred, and faster speeds.

Gait Parameters	Variables	ISI Score		
		Slower	Preferred	Faster
Pace	Walking speed	−0.184 *	−0.186 *	−0.185 *
	Stride length	−0.114 *	−0.163 *	−0.165 *
	Step length	−0.116 *	−0.162 *	−0.164 *
Phases	Double support phase	-	-	0.110 *
	Stance phase	-	-	0.105 *
GV: Pace	CV of stride length	-	0.190 *	0.156 *
	CV of step length	-	0.192 *	0.140 *
GV: Phases	CV of single support phase	-	0.116 *	0.112 *
	CV of double support phase	-	0.206 *	0.153 *
	CV of stance phase	-	0.197 *	0.185 *
GV: Rhythms	CV of stride time	-	0.190 *	0.156 *
	CV of step time	-	0.201 *	0.138 *
Bilateral coordination	GA	0.157 *	0.191 *	0.138 *
	PCI	-	0.146 *	0.164 *

CV: coefficient of variance; GA: gait asymmetry; GV: gait variability; ISI: insomnia severity index, PCI: phase coordinate index. * *p* < 0.05.

## Data Availability

The datasets generated and/or analyzed during the current study are not publicly available due to intellectual property reasons, but are available upon a reasonable request.

## Supplementary Materials

The following are available online at https://www.mdpi.com/1660-4601/17/14/5181/s1, Table S1: Raw data.
